# Embryonic and larval developmental stages of African giant catfish *Heterobranchus bidorsalis* (Geoffroy Saint Hilaire, 1809) (Teleostei, Clariidae)

**DOI:** 10.1186/2193-1801-3-677

**Published:** 2014-11-18

**Authors:** Wasiu Adekunle Olaniyi, Ofelia Galman Omitogun

**Affiliations:** Department of Environmental Biology and Fisheries, Adekunle Ajasin University, PMB 001, Akungba-Akoko, 342111 Ondo State, Nigeria; Department of Animal Sciences, Obafemi Awolowo University, Ile-Ife, 220005 Osun State Nigeria

**Keywords:** African giant catfish, *Heterobranchus bidorsalis*, Aquaculture, Oocyte, Eclosion, Fin bifurcation, Dorsal fin, Adipose fin, Developmental stages

## Abstract

The dearth of African giant catfish *Heterobranchus bidorsalis* seeds poses great threat to its aquaculture and biodiversity, hence detailed knowledge and understanding of its embryology is indispensable for its artificial propagation and conservation programmes. Photomicrographs of extruded oocyte through all developmental cell stages of live embryo to larval stage are documented with the aid of a light microscope. The optical transparency of the developing embryo enabled us to describe its deep structures, distinctive features and characterize the stages pictorially. Extruded oocyte had a mean diameter of 1 ± 0.1 mm, ~20% increase when hydrated, and bounded by double thin perivitelline membranes. The first mitotic cleavage occurred at 69 min post-fertilization (pf) resulting in 2, 4 (2 × 2 array of cells), 8 (2 × 4), 16 (4 × 4), 32 (4 × 8), 64 (2 × 4 × 8) blastomeres, then developed to morula, blastula and gastrula stages. Blastula was featured by formation of enveloping layer and yolk syncytial layer. Onset of epiboly at 3 h 57 min depicted the commencement of gastrula while closure of blastopore at 11 h 8 min marked its completion. Neurulation period was distinct from segmentation where organogenesis was fully active. Embryo sudden muscular contraction was noticed at ~17 h pf, increased prior to hatching with caudal locomotion firstly at 42 s interval. Heartbeat of embryo commenced at ~1 h before its unique eclosion at average of 72 beats/min while first larva emerged at 21 h at a controlled temperature of 28.5 ± 0.5°C. Mean total length (TL) of larvae and their pouch thickness were 5 ± 1 mm and 0.05 ± 0.02 mm respectively. 1 –day old larvae revealed 8 distinctive neuromeres and by day 3, epicanthus folds of the eyes were fully uncovered; and thereafter commenced exogenous feeding. At day 4, larvae recorded mean TL of 9 ± 1 mm and 15 caudal fin rays. The fin bifurcation to dorsal and adipose fins was observed at third and half weeks post-hatchability with the dorsal fin length to adipose fin was 1.7:1. This study, for the first time, presents significant morpho-sequential developmental stages of *H. bidorsalis* and registers its unique form of eclosion.

## Introduction

Genus *Heterobranchus* can be differentiated from other *Clariid* catfishes by the presence of large adipose fin that followed its spineless dorsal fin. The family *Clariidae* is present in African freshwater and extends to Syria, Southern Turkey and South-East Asia (Teugels 1996). Fourteen genera are recognized in the family; Teugels (1986) listed it to contain 12 African genera with 74 species and 3 Asian genera with 18 species (Teugels 1996). Genus *Heterobranchus* contains four important species namely, *H. bidorsalis* Geoffroy Saint Hilaire 1809; *H. longifilis* Valenciennes 1840; *H. isopterus* Bleeker 1863; and *H. boulengeri* Pellegrin 1922 (Reed et al. 1967; Teugels et al. 1990; Teugels 1996). The latter species presence in the genus was reported to be of great concern due to some striking different features it possesses compared with its congeners (Agnèse and Teugels 2001). Reed et al. (1967) regarded *H. isopterus* to be the smallest in the genus and very rare to be found in local waters. *Heterobranchus* and *Clarias* are the two most economic important genera in this family.

The species for this study, *H. bidorsalis* is a highly economic species that performs better than other species in family *Clariidae*. It is identified from its congeners by the presence of longer dorsal fin compared to its adipose fin with absence of black spot at its tail end. *H. longifilis* and *H. isopterus* are characterized with equal lengths of dorsal fin to adipose fin and the two can be distinguished by the absence of black spot at the end of adipose fin of latter while it is present in the former (Reed et al. 1967). The larger size of *H. bidorsalis* (length = 1.2 m; weight = 30 kg) and its congener species relative to members of the genus *Clarias* proves that the former has significant potential for aquaculture (Reed et al. 1967). It performs well in captivity by attaining maturity in 10–12 months of domestication but 2–3 years in the wild (Fagbenro et al. 1993; Adebayo and Fagbenro 2004). Also, its meat is of high quality and palatability. However, its intensive aquaculture is limited due to constraints in getting its seed from natural waters that are uneconomical and unrealistic (Adebayo and Olanrewaju 2000; Adebayo and Fagbenro 2004). Moreover, there is generally dearth of knowledge on the biology of this species, *H. bidorsalis* except fewer studies on its haematological characteristics, nutritional or feeding characteristics, salinity tolerance, digestive enzymes profile, parasite fauna and induced spawning (Fagbenro et al. 1991, 1993; Adebayo and Fagbenro 2004). This is due to the species’ limited availability, breeding constraints of longer timed sexual maturity and short breeding period which is at the peak of rainy season. Though the species has not been listed as endangered but there is risk of extinction because of environmental problems or effect of the breeding sites (Honji et al. 2009, 2012); for example, the species does not breed in the ponds but the fingerlings are sourced at the bank of large rivers (Adebayo and Olanrewaju 2000; Adebayo and Fagbenro 2004). Further threats are anthropogenic activities like the construction of dams, riparian habitat destruction, water pollution and fishing (Honji et al. 2009, 2012; Olaniyi 2014).

Limited studies have been conducted on this species for improvement on its breeding (Adebayo and Fagbenro 2004; Agbebi et al. 2005) while few studies on hybridization and growth performance in comparison with other species are just developing (Nlewadim et al. 2004; Akinwande et al. 2009; Ekelemu 2010; Oguguah et al. 2011); and this is attributed to its defined spawning season or non-spontaneous breeding system, reproductive dysfunction, poor technical/breeding knowledge, breeding and/or artificial propagation constraints and very few biological studies. Therefore, embryological studies of this species are essential for improvement on its breeding, aquaculture potentials and biodiversity. The present study aimed to investigate and describe the morphological and chronological developmental stages in the ontogeny and organogenesis of *H. bidorsalis*.

## Materials and methods

### Fish sample, selection and induced breeding

The *H. bidorsalis* broodstock were obtained from National Institute for Freshwater Fisheries Research (NIFFR), New Bussa, Niger State, Nigeria and maintained in standard conditions as described by Bromage and Roberts (1995) in the Wet Laboratory, Department of Animal Sciences, Obafemi Awolowo University, Ile-Ife, Nigeria for 2 years. The origin of *H. bidorsalis* stock from NIFFR, New Bussa, Niger State, Nigeria was from Lake Kainji, Nigeria. The induced breeding was conducted during the peak period of rainy season (June – August, 2012) that corresponds to its breeding period (Adebayo and Fagbenro 2004). Sexually matured male (Total length: 51.75 ± 3.45 cm; weight: 1.5 ± 0.1 kg) and female (Total length: 65.25 ± 5.35 cm; weight: 1.78 ± 0.17 kg) broodstock were identified and selected based on the external sexual characteristics. The matured males possessed prominent pinkish-coloured pointed genital papillae (Adebayo and Fagbenro 2004; Olaniyi and Omitogun 2013) and the females were gravid with swollen abdomen that freely oozed out eggs upon gentle pressure on their abdomen from their pinkish or reddish swollen vents (Caneppele et al. 2009; Puvaneswari et al. 2009; Honji et al. 2011, 2012; Olaniyi and Omitogun 2013).

Artificial breeding was carried out using Ovaprim® (Syndel, Canada) following standard protocols (Legendre et al. 1992; Váradi et al. 1999; Olaniyi and Omitogun 2013) with modifications (Olaniyi, 2013). Both sexes of *H. bidorsalis* were given a single dose of 1.0 ml/kg of the hormone. Induction was carried out at 27 ± 0.5°C on three sexually matured females and four males broodstock. After the latency period, eggs and sperm were obtained from the female and male broodstock respectively. The “*wet*” method of fertilization was employed whereby eggs were fertilized with diluted sperm with physiological saline solution (0.9% NaCl) (Olaniyi, 2013). Fertilized eggs were set in triplicate and incubations were carried out at a controlled temperature of 28.5 ± 0.5°C.

### Fertility, hatchability and survival

Fertility was evaluated 2–3 h (morula stage) after fertilization with respect to the total number of eggs set (equation 1) (Honji et al. 2011, 2012; Olaniyi and Omitogun 2012, 2013). The hatchability and survival percentages were calculated. The percentage hatchability (equation 2) was calculated by the number of hatched larvae (N_H_) in relation to the number of fertilized eggs (N_F_); while the percentage survival (equation 3) was calculated following Don and Avtalion (1986) and Omitogun et al. (2012) methods.
123

where;

*c* = absolute percentage of fertile eggs;

*i* = initial number of the eggs set;

*n* = number of eggs which survived up to a given developmental stage;

N_H_ = number of larvae hatched;

N_F_ = number of eggs fertilized.

### Post-Induction management

Water parameters such as pH, alkalinity, dissolved oxygen and temperature were monitored, while the optimum oxygen level was maintained with RESUN LP- 100 low noise air-pump following Olaniyi and Omitogun (2012). After yolk absorption, the fry were fed *ad libitum* with *Artemia naupli* shell free feed, 54% CP (INVE® Aquaculture Nutrition, Ogden, Utah, USA) following Howell et al. (1998) and Honji et al. (2012). Feed was made available at all times by feeding at short intervals like eight (8) times daily.

### Embryonic studies and photomicrography

Ten (10 ± 2) fertilized oocytes were randomly sampled into Petri dish and their developments were studied under the microscope. The development stages were also verified with the ongoing stages in the incubation tanks. Oocyte, embryonic and larval developmental stages were studied right from unfertilized matured oocyte, to fertilization and hatching with the aid of biological computer-aided light microscope (Olympus Trinocular Microscope XSZ-156 T) mounted with digital camera (DCM 130, Res 1.3 M pixels). With this microscope, developing oocytes, hatching, alevin and larval stages were viewed, monitored and photographed and their times of occurrence were documented.

### Interpretation of the terminologies

The terminologies used for the developmental stages right from unfertilized matured oocyte, to fertilization, hatching and post-hatching are in line with earlier embryonic developmental studies in order Siluriformes and other fish species’ orders (Cardoso et al. 1995; Kimmel et al. 1995; Caneppele et al. 2009; Honji et al. 2012; Olaniyi and Omitogun 2013).

## Results

### Sample selection and induced breeding

Ovulation was achieved within the latency period of 14 ± 1 h at ambient temperature (27 ± 0.5°C). Mean spawned unfertilized eggs measured 1 ± 0.1 mm diameter and had about 20% increase in size when hydrated (1.2 ± 0.2 mm). Water quality parameters such as pH, alkalinity, dissolved oxygen and temperature at incubation were 7.1 ± 1, 112.31 ± 1.14 mg/l, 24.5 ± 0.5 mg/l and 28.5 ± 0.5°C respectively. All the broodstock induced to spawning responded well to artificial breeding and had 100% survival. The mean spawned egg was 55.3 ± 7.3 g. The percent fertility, hatchability and survival revealed 80.67 ± 6.03, 68.33 ± 3.06 and 61.00 ± 1.73 respectively.

### Characterization of the stages of development

It is very important to note that the following established stages are dependent on different kinds of species and the conditions of the experimental set-up, for example the temperature. The stages, as used in this study, are explained thus:

#### Oocyte stage

This is an unfertilized matured egg characterized with spherical or ovoid shaped structure and largely filled yolk content. It is not regarded as part of embryonic development stage when not fertilized.

#### Embryonic stage

This period starts immediately when the oocyte is fertilized till the embryo hatched out of the chorion.

#### Post-embryonic stage

This period of development starts immediately when the larvae hatched out of chorion till the complete yolk absorption.

#### Larval stage

This stage commenced after the complete yolk absorption and the larvae started exogenous feeding. This is the early larva period whereby the hatched larva has completed most of its morphogenesis and starts to grow rapidly. This stage can last for few days or months.

### Developmental stages and photomicrography

**Figure 1:**
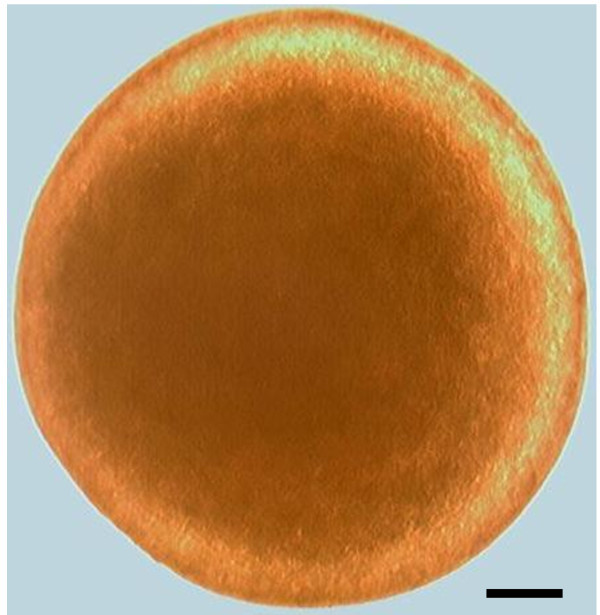
**Photograph of oocyte stage of an unfertilized** ***Heterobranchus bidorsalis*** **egg.** Scale bar: 1 mm; Magnification: ×40.

**Figure 2:**
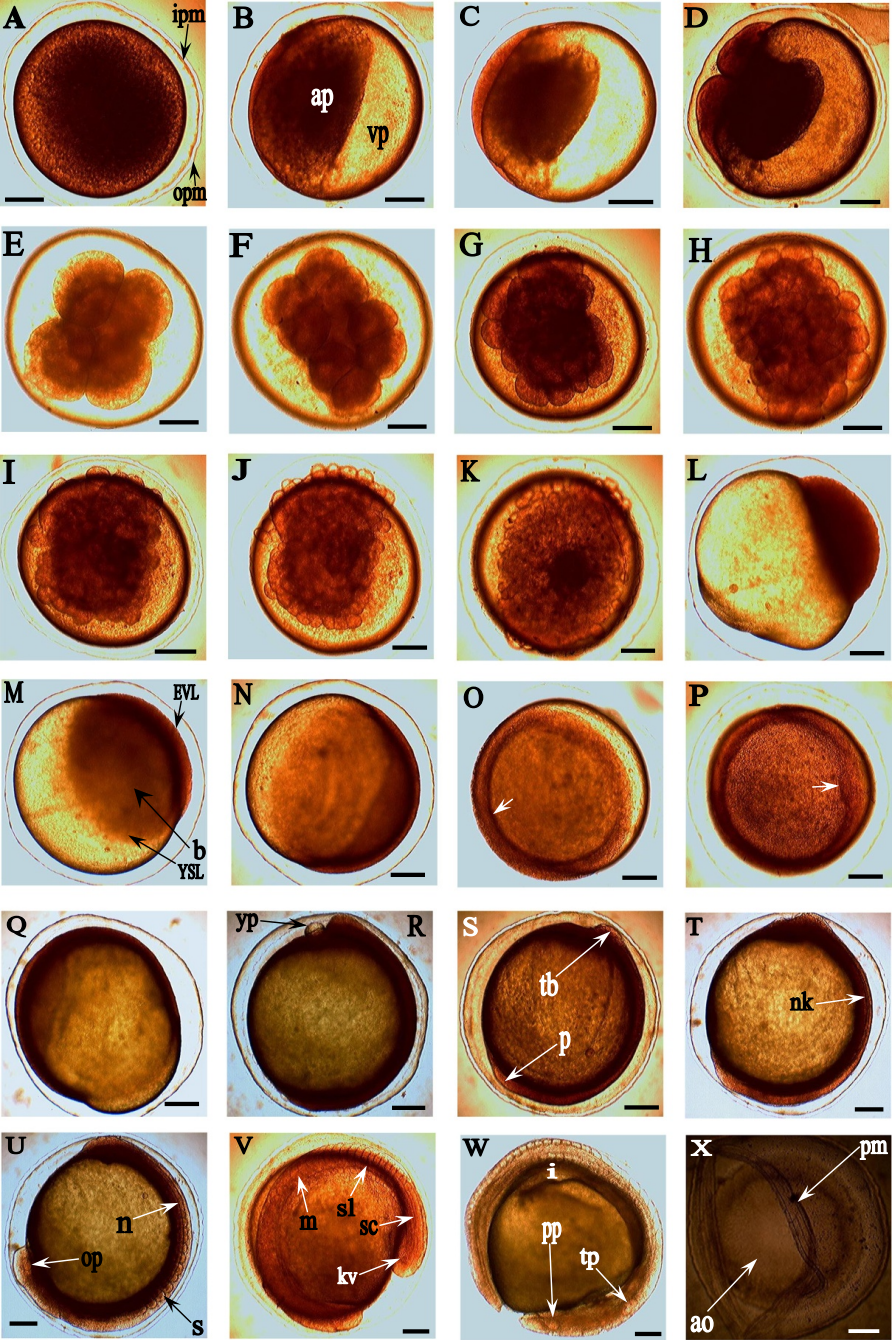
**Early embryonic developmental stages of** ***Heterobranchus bidorsalis*** **. A**: Fertilized egg; outer perivitelline membrane, opm; inner perivitelline membrane, ipm. **B**: Polar division. Anal pole, ap; Vegetal Pole, vp. **C**: One-cell stage. **D**: Two-cell stage. **E**: Four-cell stage. **F**: Eight-cell stage. **G**: Sixteen-cell stage. **H**: Thirty-two-cell stage. **I**: Sixty-four-cell stage. **J**: Morula. **K**: Blastula (High). **L**: Blastula (low). **M**: Enveloping layer, EVL; yolk syncytial layer, YSL; blastoderm, b. **N**: 50% Epiboly. **O**: Germ ring (shown by arrow). **P**: Embryonic shield (shown by arrow). **Q**: 75% Epiboly. **R**: 95% Epiboly; yolk plug, yp. **S**: Epiboly completed (100%); polster, p; tail bud, tb; perivitelline membrane thickens. **T**: Neural keel, nk; Tail bud expansion noticed. **U**: Formation of somite blocks; somite block, s; notochord, n; early emergence of optic primordia, op. **V**: Kupffer’s vesicle, kv; spinal cord, sc; sclerotome, sl; myotome, m. Earlier formed somites developed into myotome blocks and progressed caudally. Differentiation of aggregated tail bud cells forming notochord's primordia and spinal cord, marking pharygula stage; somites develop and mature cephalocaudally. **W**: Optic placode, pp; and otic placode, tp; with the two tiny otoliths; inward cellular movement, i, noticed. **X**: Hollowed embryonic membrane/pouch during hatching; chorion or perivitelline membrane, pm; area of emergence, ao. Scale bar: 1 mm; Magnification: ×40.

**Figure 3:**
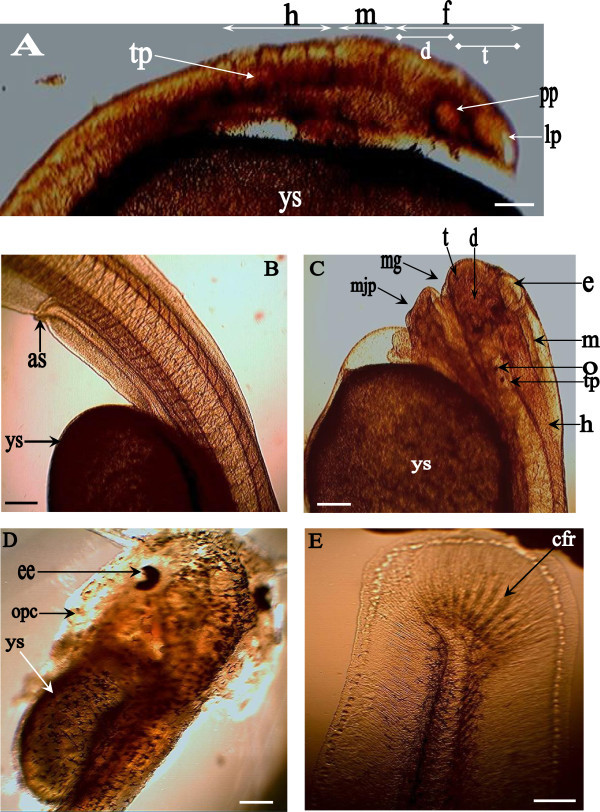
**Post-embryonic and larval developmental stages of** ***Heterobranchus bidorsalis*** **. A**: Cerebellar differentiation. Hind brain, h; mid brain, m; fore brain, f (diencephalon, d; telencepahlon, t); otic placode, tp; optic placode, pp; olfactory placode, lp; yolk sac, ys. **B**: First day old larva. Anus, as; yolk sac, ys. **C**: Second day old larva. Telencephalon, t; diencephalon, d; epiphysis, e; mid brain, m; hind brain, h; otolith, o; otic placode, tp; mouth gape, mg; mouth and jaw primordia, mjp; yolk sac, ys. **D**: Third day old larva. Yolk sac, ys; eye, ee; operculum, opc. **E**: Fourth day old larva. Caudal fin rays formation; caudal fin ray, cfr. Scale bar: 1 mm; Magnification: ×40.

The development of embryos (~87%) in the experimental set-up tanks corresponded to the developmental stages of the randomly sampled fertilized oocytes. Tables 1 and 2 show the summarized developmental stages of *H. bidorsalis* with the average timing at early embryonic and post hatchability (post-embryonic and larval) periods respectively. Figure 1 depicts the photomicrograph of matured unfertilized egg, while Figures 2 and 3 show the sequential developmental stages from fertilized egg to early embryonic developments and post embryonic developments to exogenous feeding or larval levels, respectively. All the accompanying photographs are of living specimen.

**Table 1:** **Early embryonic developmental stage of** ***Heterobranchus bidorsalis*** **at 28.5 ± 0.5°C**

Stage	Time from fertilization (min)	Main events
Zygote	0	Concentration of yolk at the centre; evident jelly coat; increase in perivitelline space (Figure 2A).
Animal and vegetal poles	45	Formation of embryonic disc; distinct vegetal pole; pigmentation of animal pole (Figure 2B).
One-cell	57	Bulging of protoplasm at animal pole (Figure 2C).
Two-cell	69	First mitotic cleavage; discoidal meroblastic division into two equal size blastomeres (Figure 2D).
Four-cell	84	Second mitotic cleavage; meridional division at right angles; 2 × 2 array of blastomeres (Figure 2E).
Eight-cell	94	Cleavage of earlier four-cell blastomeres; division similar to the first mitotic cleavage resulting into two parallel rows of four blastomeres each; 2 × 4 array (Figure 2F).
Sixteen-cell	104	Formation of sixteen blastomeres; 4 × 4 array (Figure 2G).
Thirty-two cell	114	Formation of thirty-two blastomeres; 4 × 8 array (Figure 2H).
Sixty-four cell	127	Irregular cell divisions; overlapping of subsequent cleavages; 2 × 4 × 8 array (Figure 2I).
128 cell	137	Irregular cleavages produce unequal blastomeres of reduced sizes, leading to morula stage (Figure 2J).
Morula	161	Irregular cell divisions; numerous blastomeres appearing like ‘mulberry’ (Figure 2J).
Blastula	165	Flattening of blastodermal cells resulting into two distinct layers, viz: high and low blastula (Figure 2K and L); formation of enveloping and yolk syncytial layers; evident blastoderm (Figure 2M).
Gastrulation	237 (3 h 57 min)	Expansion of blastoderm; epiboly commenced (Figure 2N – 2Q); formation of germ ring; embryonic bud discernable (Figure 2O); thickening of embryonic shield and perivitelline capsule (Figure 2P); increase in foul and offensive odour.
	Late (completed) = 668 (3 h 57 min)	
Neurulation	669	Neural plate discerned as thickened structure; formation of neural groove delineating the neural keel (Figure 2T); notochord development (Figure 2U).
Segmentation	Early = (1st block observed at) 674	Formation of somite blocks; body pigmentation developed dorso-cephalocaudally (Figure 2U and V). Formation of cranial, dorsal, ventral and caudal regions (Figure 2W).
	Late = (completed with the formation of other body organs/organelles) = 979	Early organogenesis.
Optic/auditory buds/Otic placode		Development of otic placode into otic vesicles depicting two tiny otoliths (Figure 2W); differentiation of notochords.
Pharygula period		Development of somites to myotome blocks; formation of sclerotomes; well differentiation of notochord depicting pharygula stage; somites matured (Figure 2U and W).
Caudal bud		Pigmentation continues; somite blocks are more closely packed and well aligned (Figure 2W).
Muscular contraction	1042	Caudal-locomotion commenced at average of 42 s.
Heart-beat	1243	Heart beat commenced at average of 72 beats/min; pigmentation of the point of the heart, eye and other parts increased (Figure 2V and W).
Hatching	1278 (21 h)	Muscular contractions of myotome blocks increased; lashing of the detached and free caudal end against chorion; successful hatching (Figure 2V and W); unique emergence of the newly hatched larvae from the hollowed chorion (Figure 2X).

**Table 2:** **Post-embryonic and larval developmental stages of** ***Heterobranchus bidorsalis*** **at 28.5 ± 0.5°C**

Stage	Main events
Early hours (<12 h post hatch)	Recently hatched larvae are translucent; possessed slightly curved body; characterized with progressive cerebellar morphostructural differentiation revealing the telencephalon, mesencephalon and rhombencephalon; otic placodes are located at the basal end of the rhombencephalon (Figure 3A).
First day	Head protruded; heart, lens placode and otic vesicles are evident; olfactory placodes are delineated (Figure 3A); blood circulation commenced; tail straightened out and head lifted dorsal-upward; evident mouth primordia; end spot of cellular inward movement forms the anus (Figure 3B).
Second day	*Early hours:* Sealed mouth; diminutive club-shaped barbell; head lifted up and gradually separating from the yolk sac; otic vesicle shifted closer to the lens placode (Figure 3C).
	*Late hours:* gaping of the mouth in direct relation to partly raising of eyes epicanthus; increase in barbell length; further development of alimentary canal. Increase in separation of head away from yolk sac; development of jaw cartilage led to increase in mouth opening; melanopohore formed around the cranial region; pigmentation increase around the eyes (Figure 3C).
Third day	Melanophores spread cephalocaudally; reduced yolk sac; eyes epicanthus uncovered (Figure 3D).
Fourth day	Slightly opaque larvae; blood inter-circulating the caudal fins; epicanthus fold totally uncovered. Larvae were photophobic; fin bifurcation to dorsal and adipose fins not yet occurred; formation of caudal fin rays (Figure 3E).

### Stages at oocyte and embryonic developmental phase

#### Oocyte

The matured oocyte is a spherical shaped structure with largely filled yolk content. It is light greenish-brown, elliptical, adhesive, demersal, and oil-globuless structure. It measured 1 ± 0.1 mm in diameter (Figure 1).

#### Fertilization/Zygote formation

The yolk content gathered and shrank to the centre immediately following a successful fertilization, and the oocyte became more adhesive in nature. Moreover, the jelly coat became more evident and the perivitelline space secured more significant area. The fertilized oocyte revealed double thin membranes (Figure 2A).

#### Polar divisions

Progressive oocyte development led to distribution of its content into two major poles, viz: animal and vegetal poles (Figure 2B). This stage was featured by the thickening of the perivitelline membrane. The animal pole (germ pole) consists of pigment granules (nucleus and cytoplasm) while the vegetal pole (vitelline part) is largely yolky. The vegetal pole was diametrically opposite of the animal pole. During the distribution, the animal pole continuously became more pigmented with non-yolky cytoplasm and gradually bulging up to form the next stage, one-cell stage; while the vegetal part became clearer and translucent. This is referred to as 'Polar division'. This stage was visible to the naked eye whereby the non-yolky cytoplasmic germ pole was characterized by a red spot at an end of the fertilized ovum. The red spot feature became more evident as the development progressed.

#### One-cell stage

There was a protoplasmic bulge at the animal pole depicting one-cell stage (Figure 2C). The segregation of the non-yolky cytoplasm at the animal pole was continuous making it more pigmented; thereby separating the blastodisc away from the vitelline parts and made it more translucent. This stage was also featured by the thickening of the perivitelline membrane.

#### Two-cell stage

This is the first mitotic cleavage. The protoplasmic bulge of one-cell stage divided in a discoidal meroblastic pattern and resulted in the formation of two equally-sized blastomeres (Figure 2D). The cleavage was meridional and restricted only to the animal pole.

#### Four-cell stage

This is the second mitotic cleavage. The division was meridional but at right angles to the first cleavage. The furrow cut through the existing blastomeres in a discoidal division (2 × 2 array) at the longer axis of the ellipsoidal shape and resulted to four blastomeres of equal sizes (Figure 2E).

#### Eight-cell stage

This is the third mitotic division. The division was similar to the first mitotic cleavage, but operated on the four blastomeres; that is, two meridional clefts occurred simultaneously on the earlier 2 × 2 array blastomeres. The clefts are parallel to the first furrow established on the one-cell staged blastodisc that firstly gave two-cell stage. This resulted to ellipsoidal structure of two parallel blocks of palindromic four blastomeres each (2 × 4 array) giving eight blastomeres (Figure 2F).

#### Sixteen-cell stage

This is the result of the cell cleavage from the earlier paired four-cell blastomeres. The cleavage, fourth mitotic division, was similar to the second mitotic cleavage. Two new furrows were established parallel to the earlier second mitotic division thereby resulting to 4 × 4 array blastomeres (Figure 2G).

#### Thirty-two-cell stage

The fifth cleavage resulted in 4 × 8 arrays making 32 blatomeres. These cleaves were irregular and not smooth as the earlier furrows. The blastomeres are not well aligned like the previous stages (Figure 2H).

#### Sixty-four-cell stage

This is the sixth form of cleavage that resulted in 64 blatomeres. The cell divisions were very high, irregular and did not follow the earlier pattern. The array presented 2 × 4 × 8 arrangement, that is presentation of two cell-layers of 32 blastomeres (4 × 8 arrays) giving rise to 64 blatomeres (Figure 2I). The earlier or superior cells gave rise to the superficial blastomeres. The resulting blastomeres are in small sizes.

#### Morula stage

There were irregular but continuous cell divisions producing tiers of numerous cells of reduced sizes. Cell proliferation reached a stage that counting was impossible; the cells were highly compacted, mounting on top of another, and appeared like 'mulberry' (Figure 2J).

#### Blastula stage

The cleaved blastomeres were highly compacted and heavily consolidated forming deeply coherent cells, blastoderm, on top of large yolk cells. At this stage, two blastula levels were noticed viz; high and low. There was cell differentiation from high blastula (Figure 2K) to low blastula (Figure 2L) levels, descending from the high structured to low formation. The blastula (low) stage was characterized by flattening of the blastodermal cells whereby compacted cells were condensed to form blastoderm (Figure 2M). During the transitional stage from high blastula to low blastula, the condensed cellular materials formed an epithelial layer that is, enveloping layer (EVL) which covered the underlying blastomeres. Adjacent to the blastoderm is the formation of a cellular structure - yolk syncytial layer (YSL) (Figure 2M). The YSL was formed at the fusion of blastomeres marginal layer to the yolk cell. Then, the EVL started spreading out, moving stealthily and gradually extending towards the vegetal parts, marking the onset of epiboly. The onset of epiboly depicted the transitional stage from the low blastula to the onset of gastrula.

#### Gastrulation

The expansion of blastoderm and slow peristaltic movement of the cellular perivitelline fluid were noticed. This is the commencement of epiboly - that is the very slowly morphogenetic peristaltic cellular movement. This movement involves more or less contraction and expansion of the blastoderm. The movement later increased gradually and consistently. At about 50% epiboly (Figure 2N), there was the definitive germ ring (Figure 2O) evident around the blastoderm. The embryonic shield was discernible with thickness of the chorionic/perivitelline capsule (Figure 2P). The characteristic cellular inward movement continued (Figure 2Q) until the blastopore was closed marking the end of epiboly and/or gastrulation. Prior to the completion of epiboly was the protrusion of yolk cell beyond the blastoderm margin referred to as the yolk plug (Figure 2R). Tail bud was noticed forming at 95% epiboly (Figure 2R); and there was eventually a completed epiboly (Figure 2S) revealing the polster and the tail bud. This stage was highly characterized with offensive odour.

#### Neurulation

The neural plate was discerned as thickened structure along the embryonic axis, and formed neural groove delineating the neural keel (Figure 2T). This resulted to formation of rodlike structure, notochord, at the dorsal part of the developing embryo (Figure 2U). It indicated the dorsal part location of the forming embryo and the supporting vertebrae column. This was viewed along the prospective head region marking out the polster. The tail bud bulged expansion was also noticed.

#### Segmentation

Formation, development and maturity of somite blocks, and the body pigmentation started at the cephalic parts of the embryo and progressed caudally. The cephalic and caudalic presentations were identified based on cell differentiations and their developmental processes into some primary organs. For example, observation of the polster and auditive parts at the anterior axis revealed those parts as the cranial/cephalic region (Figure 2U) while appearance of the kupffer's vesicle designated its area of occurrence as the caudal/posterior region (Figure 2V). The prominent tail bud deeply revealed the kupffer's vesicle that later disappeared during the development.

#### Pharyngulation

The somite blocks were closely packed and well aligned giving more strength to the developing embryo. Somites developed into myotome blocks that are concave in shape towards the posterior. This development commenced from the early formed somites and progressed caudally. It shows clearly when viewed from the early formed somites and the forming sclerotomes within them. The translucent nature of the larvae made their cell differentiations well observed revealing notochord and spinal cord's primordia cells differentiation. The notochord and spinal cord are discernible marking pharyngula stage while developments continue cephalocaudally following somites maturity. Body pigmentations are now more prominent (Figure 2V). At this stage, the embryo's first sudden muscular contraction was noticed approximately 17 h post-fertilization (pf).

#### Optic, otic placodes and auditory buds

Optic primordium was discernible. Embryonic keel/shield was thickened and more somite blocks were still forming. At the anteriodorso-cephalic region of the developing embryo, the otic placode developed into otic vesicles with the early emergence of two (2) tiny otoliths. The caudal end detached gradually and frees itself totally from the blastodisc. Somites blocks were more closely packed and well aligned. The end spot of cellular inward movement (where blastopore closed) was apparent at the antero-caudal region moving towards meta-ventral region of the embryo as development progressed (Figure 2W).

#### Hatching

Before eclosion of the encapsulated embryo, there were vigorous muscular contractions by the embryo that consist of radial and rotational movements especially at the caudal parts. The caudal-locomotion subsequently occurred at mean interval of 42 s, and the contraction increased as the development progressed. The contraction was aided by the well-aligned consolidated muscular blocks of myotomes and supported by sclerotomes (Figure 2V and W). The heartbeat commenced at the average of 72 beats/min. There were twitches and vigorous lashings of the detached free - end of caudal part against the chorion. The twitches and lashings became progressive, stronger and frequent. These facilitated the hatching. The first larva hatched at 21 h while others follow. The newly hatched larvae emerged from the hollowed chorion (pouch) firstly with the caudal part and left a thick pouch behind (0.05 ± 0.02 mm thickness) as shown in Figure 2X. Hatchability occurred within 30 min to 1 h from the first hatched larva. The hatched larva measured average total length of 5 ± 1 mm.

### Stages during post-embryonic and larval developmental phases

#### First day old larva

Recently hatched larvae were translucent, curved and characterized with progressive cerebellar morphostructural differentiation revealing the telencephalon and diencephalon that made the forebrain, mesencephalon (midbrain) and rhombencephalon (hindbrain) (Figure 3A). The olfactory placodes were overlying the telencephalon, the otic placode were located at the basal end of the rhomboncephalon while the larvae carried largely filled spherical yolk sac.

As development progressed during the first day (24 h) of hatch, the head was protruded and body pigmentation continued. The heart, lens placodes, otic vesicles, olfactory placodes were noticed, while the mouth was yet to differentiate. No diminutive barbell was observed, and onset of blood circulation was noted at mean duration of 9 h post hatch (ph). As the posterior body segment straightened out, the head also lifted dorsally-upward. No dorsal and adipose fins were observed. The spot of invagination that moved towards the meta-ventral region of the embryo was observed forming the anus or urogenital vent (Figure 3B).

Newly hatched larvae are soft tissued, fragile and helpless. As the larvae are being hatched, organogenesis was still actively ongoing at the cephalic parts especially at the cranial region. The earlier segmentation of the anterior cranial part revealed about eight (8) distinctive ridges called neuromeres. The first three (3) neuromeres from anterior cranial part are larger. The first two (2) ridges, telencephalon and diencephalon, formed the fore brain while the last larger ridge made the mid brain, mesencephalon. The latter five (5) neuromeres made the rhombomere (hind brain segment). The olfactory placode was seen overlying the telencephalon while the otic placode was observed overlying parts of third and fifth rhombomere and full part of fourth rhombomere (Figure 3C).

#### Second day old larva

At the early hours of the second day the following were observed: sealed mouth, diminutive club shaped barbell, head lifted up and separating from the yolk sac and the otic vesicle has shifted closer to the lens placode. By the mid hours, the mouth primordia were partly gaped opened. The late hours of the second day recorded the mouth ajar, raised epicanthic fold of the eyes, increase in appendage barbell length and development in alimentary system. Rapid development of the mouth and jaw primordia was noticed. These developments gradually led to the forward uplift of the head that was protruded anterio-dorsally upwards thereby separating it away from the yolk sac. The cartilage development in the jaw was significant leading to more advance opening of the mouth. Few melanophores were noticed around the cranial region, and more pigmentation was observed around the eyes (Figure 3C).

#### Third day old larva

Melanophore had spread cephalocaudally and covered the body parts but concentrated heavily on the cranial region. Epicanthus fold the eyes was fully uncovered. By the late hours, the yolk sac content had reduced significantly and the mean total length of the larvae was 8 ± 1 mm (Figure 3D). Some larvae were observed feeding on the artemia offered.

#### Fourth day old larva

The translucent nature of the larvae had reduced to slightly opaque. Though, blood circulation could still be visualized clearly within the body depth as it was inter-flowing within the caudal fins. There were no more epicanthus covering the eyes and the iris were clearly shinning and reflective (Figure 3D). The larvae were observed to be photophobic and preferred darkness. Operculum was seen flapping the branchial arch of the larvae and the alimentary system are now well developed. About fifteen (15) caudal fin rays were observed in the forming caudal fins (Figure 3E). The mean total length of the larvae measured 9 ± 1 mm. There was early differentiation of dorsal and anal fins but no fin rays were observed. The dorsal and anal fins were sparsely pigmented and transparent. No bifurcated fin was observed at this period. The fin bifurcations to dorsal and adipose fins could only be noticed at the third and half weeks of post-hatchability with the dorsal fin longer than the adipose fin (dorsal fin length: adipose fin length =1.7:1). Moreover, no black spot was observed at the tail end of the adipose fin. The larvae mortality due to cannibalism was found to be insignificant.

## Discussion

The embryogenesis of *H. bidorsalis* is a short duration similar to other total spawners species that exhibit reproductive migration and spawns either adhesive eggs as in *H. longifilis* and *Clarias gariepinus* (Legendre and Teugels 1991; Olaniyi and Omitogun 2013), *Rhinelepis aspera* (da Rocha et al. 2009) or non-adhesive eggs in *Pseudoplatystoma coruscans* (Lamas 1993; Cardoso et al. 1995), *Rhamdia hilarii* (Godinho et al. 1978) and *R. quelen* (Pereira et al. 2006). This study provides information on fish handling of *H. bidorsalis*, morphology of its oocyte, embryonic, and larval developmental stages focusing on its sequential and chronological details as established with their photomicrographs.

### Sample selection and induced breeding

The mean spawned egg of *H. bidorsalis* was small in relation to other catfish species (Caneppele et al. 2009; Honji et al. 2012) and this revealed that the species exhibits reproductive dysfunction in captivity (Mylonas et al. 2010) like some teleost species such as *P. fasciatum* (Leonardo et al. 2004), *P. corruscans* (Campos 2005), *R. quelen* (Baldisserotto and Neto 2005), *Steindachneridion melanodermatum* (Ludwig et al. 2005), and *S. parahybae* (Honji et al. 2009, 2012). The survival (100%) of the broodstock after spawning showed that the species have good promise for artificial propagation. The fertility of eggs rests on fecundity, egg size and quality (Bromage and Roberts 1995; Olaniyi and Omitogun 2012, 2013). The hatchability is dependent on the significant effect of water quality parameters, for example, the temperature; that is, the developmental process from fertilized egg to hatching like all other biological processes is dependent upon temperature. The higher the water temperature, the faster the eggs are hatched and the better the survival (Haylor and Mollah 1995; de Graaf and Janssen 1996; Olaniyi and Omitogun 2012, 2013). The full detail of the induced spawning of *H. bidorsalis* has been discussed elaborately in a forthcoming article.

### Oocyte development

The double thin perivitelline membranes provide stability to the embryo (Winnicki et al. 1970; Korzelecka-Orkisz et al. 2010; Honji et al. 2012), while the perivitelline space that was filled with protective fluid cushions the egg and embryo from external injury (Khan 1972; Puvaneswari et al. 2009; Buzollo et al. 2011). Olaniyi and Omitogun (2013) reported “a thin perivitelline membrane” but actually showed and explained double thin membranes of the perivitelline. Shardo (1995) reported that the combination of chorion (perivitelline membrane) and the filled perivitelline space function as mechanical protection especially in running water, osmotic regulation, and prevention of polyspermy (Buzollo et al. 2011). The gelatinous coat that encapsulated the egg is a characteristic of Silurifomes, a form of protection for the egg whether adhesive or not (da Rocha et al. 2009) and for attachment to substratum. The adhesive and non-adhesive natures of Siluriformes egg explain the chemical composition of the gelatinous coat (Rizzo et al. 2002; da Rocha et al. 2009). Eggs of *H. bidorsalis* are same or close in size to a sister species, *C. gariepinus* that recorded 0.9–1.1 mm (Olaniyi and Omitogun 2013), 1–1.2 mm (de Graaf and Janssen 1996); but smaller to other catfish species *Silurus glanis* L. that ranged 1.4–2.5 mm (Korzelecka-Orkisz et al. 2010). However, variation in egg size can be due to strain and this is one of the characteristics (Thakur 1980; Puvaneswari et al. 2009) that determines broodstock egg quality (Bromage and Roberts 1995).

### Embryonic development

The cleavage of *H. bidorsalis* oocyte demonstrated discoidal meroblastic pattern of telolecithal egg (Kimmel and Law 1985; Kimmel et al. 1995; Hall et al. 2004; Buzollo et al. 2011). This kind of division is incomplete and exclusively occurs in the animal pole, whereby sister cells partially separated from one another by cytokinesis in all the early cleavages, and along the blastodisc margin in late cleavages and early blastula periods (Balinsky 1970; Kimmel and Law 1985; Kimmel et al. 1995; Leme dos Santos and Azoubel 1996; Hall et al. 2004; Buzollo et al. 2011; Olaniyi and Omitogun 2013). The pattern of egg division in vertebrates is dependent on amount of yolk, its distribution and proportion it occupies with respect to the cytoplasm that will constitute the blastodisc (Gilbert 1991; Cardoso et al. 1995). The cleavage pattern from onset was synchronous, regular and well aligned up to approximately the sixth cleavage though many of the blastomeres were completely mounted themselves at the sixth cleavage Beyond this stage, the irregularity of cleavage became more pronounced and asynchronous such that the numerous blastomeres produced are referred to as “mulberry” or “half-berry” or “ball” like shape (Honji et al. 2012; Olaniyi and Omitogun 2013) due to some buried cells, or deep cells that were covered or mounted by tiers of cells that gave rise to the EVL of blastodisc (Kimmel et al. 1995). This period is termed “blastula”. These features of blastula significantly occurred at eighth zygotic cycle or 128–cell stage before the onset of gastrulation (Kimmel et al. 1995). The beginning of cellular movement which may be invagination, involution or ingression, or the spreading out of EVL towards the vegetal parts that marked the onset of epiboly indicated transitional stage from (low) blastula to the onset of gastrula (Kimmel et al. 1995; Leme dos Santos and Azoubel 1996; Buzollo et al. 2011). The condensed cells are referred to as *“convergent extension”*. The convergence is the movement of hypoblast and epiblast cells towards the future dorsal side of the embryo while the extension involves the elongation of anterio-posterio axis (Rhode and Heisenberg 2007). The EVL is a collective structure that serves as an anchorage or protective sheath, overlays and binds the flattened blastomeres of the blastula (low) (Koppen et al. 2006; Rhode and Heisenberg 2007) while the YSL determines the cells fate in the overlying blastomeres (Koos and Ho 1998). Moreover, only EVL does not take part in convergent extension process but got distributed over embryonic surface and later becomes periderm (Kimmel and Warga 1987; Kimmel et al. 1990; Rhode and Heisenberg 2007).

The completion of gastrulation was marked by the closure of blastopore (Kimmel et al. 1995; Buzollo et al. 2011), but Kimmel et al. (1995) reported that it is likely to continue at the tail bud because of the movement of deep cell layer from one stage to another. However, the formation of the embryo, like in annual fishes, fully commenced after epiboly had completed (Wourms 1974). Thereafter, the polster and tail bud were demarcated and these are good morphological markers for the identification of the cranial and caudal parts of developing embryo respectively (Olaniyi and Omitogun 2013). The perceived typical characteristic foul odour was due to longer time staged and high developmental processes at gastrula.

In this study, the end spot of cellular inward movement formed the anus or urogenital vent at the meta-ventral region of the embryo. However, series of staging and sectioning of cells is necessary to really study deeper into the cells (Wood and Timmermans 1988; Kimmel et al. 1995), to reveal the cellular movements if it is invagination, involution or ingression. Kimmel et al. (1995) reported no blastocoele during blastula, no archenteron at gastrula and no blastopore formations in zebrafish *Danio (Brachydanio) rerio* developmental processes. Buzollo et al. (2011) indicated the presence of pseudoblastocoele – that is the appearance of irregular spaces among the blastomeres, in *Pimelodus maculatus*. Kimmel et al. (1995) had earlier reported these irregular spaces among the blastomeres during the embryo development of *D. rerio* and did not regard it as real blastocoele (that is the formation of a large space or cavity between the yolk and the blastomeres) but rather suggested the blastula period to be referred to as *“stereoblastula”*. Some embryological studies in teleostean species (Trinkaus 1984; Ganeco 2003; Marques 2008) have also reported presence of these irregular spaces among some blastoderm cells and referred to them as blastocoel with no disparity (Buzollo et al. 2011).

This study revealed distinct neurulation and segmentation periods in *H. bidorsalis* like in the embryonic development of amphibian, but in contrary to *D. rerio* that showed overlapping periods and no distinct neurulation (Kimmel et al. 1995). The organogenesis fully occurred during segmentation period whereby the early segmentation stage was characterized with formation of somite blocks, cephalic region featured by polster and auditive parts, caudal region featured by kupffer's vesicle. These organs delineated the embryonic axis showing the cranial, dorsal, ventral and caudal regions (Kimmel et al. 1995; Buzollo et al. 2011; Honji et al. 2012). The late segmentation was characterized by closely packed and well aligned somite blocks leading to the formation of myotome muscle blocks, well delineation of the end spot of cellular inward movement (where blastopore closed) at the antero-caudal region forming the anus at the meta-ventral region, commencement of heart beat, presence of free tail or caudal part that leads to muscular contraction and/or caudal locomotion (Kimmel et al. 1995; Buzollo et al. 2011; Honji et al. 2012; Olaniyi and Omitogun 2013). The features of embryo at the pharyngula stage are entailed in the segmentation period. The “pharyngula” as depicted in this study, is the embryonic phylotypic stage, at which the embryo develops features that defines it as a vertebrate (chordate), possessing the notochord, neural tube, pharyngeal arches, somites and postanal tail (Ballard 1981; Kimmel et al. 1995). Other features are, the morphogenesis - when the posterior body segment straightened out and the head lifted dorsally-upward, pigmentation of the body, onset of blood circulation and the fins formation, and finally the uncoordinated individual myotome flexions leading to rhythmic bouts of swimming (Kimmel et al. 1995). The revealed kupffer's vesicle is an important feature of teleost embryo (Cardoso et al. 1995), and indicate the allantoic rudiment (Kimmel et al. 1995). The cells at animal pole formed the cranial region; convergence and extension ended up in dorsal part while the tail bud cells formed the posterior or caudal part. The formation of otics and otolith developed to essential auditory structures (Kimmel et al. 1995).

*H. bidorsalis* form of hatching is unique because the embryo emerged with its caudal part through the pouch or hollowed membrane unlike other order Siluriformes member e.g. *C. gariepinus* that breaks the chorion into granules during hatching (Olaniyi and Omitogun 2013). The hatching was facilitated by the tightly packed myotome blocks of the somite trunk that generated muscular twitches or contractions especially at the caudal part (Honji et al. 2012; Olaniyi and Omitogun 2013). The heart beats (respiration) ensured more strength into the embryo, while the vigorous lashes of the free caudal end against the chorion facilitated successful hatching; however weaker embryos are expected not to hatch or emerge from the hollowed chorion. This mode of hatching is of aquacultural significance because the pouch or hollowed membrane will not decay early; thereby contributing tremendously to water pollution and turbidity that will cause greater oxygen depletion and high mortality of the larvae. The hatching time of *H. bidorsalis* in this study at 21 h at 28.5 ± 0.5°C was earlier than that obtained by Adebayo and Fagbenro (2004) at 24 – 28 h for the same species but at 27 ± 1°C. This difference is due to temperature difference; that is, the higher the temperature, the better and faster the hatchability (Hogendoorn and Vismans 1980; Galman and Avtalion 1989; de Graaf and Janssen 1996; Legendre et al. 1996; Buzollo et al. 2011; Olaniyi and Omitogun 2012, 2013). The hatching period that occurred within 30 min to 1 h from the first hatched larva was dependent on the fertilization period within the oocyte; hence, the faster the fertilization within the oocyte, the better the hatching period and the survival of the embryos.

### Post-embryonic and larval development

During hatching of the eggs, water pollution and turbidity increased continuously, and consequently, microorganisms were produced progressively from the pouches or hollowed membranes, non-viable, unhatched and dead eggs. The newly hatched larvae are predisposed to predatory attacks due to their fragility and helplessness, because they lack parental care as one of the challenges of the species they belong (da Rocha et al. 2009). Therefore, prompt and adequate management is important to achieve optimal hatching and survival. The water temperature for this study (28.5 ± 0.5°C) confirmed the recommended optimal temperature between 25°C and 29°C for *H. bidorsalis* congener, *H. longifilis,* (Legendre and Teugels 1991) by which high hatching could be achieved; moreover, prolonged development due to low temperature (Adebiyi et al. 2013) leads to death of developing embryo. Also, constant supply of fresh water in a trickling mode for flow-through system to wash away impurities that can lead to pollution or turbidity of incubation water, and sufficient dissolved oxygen are indispensable at this phase to achieve successful breeding.

The number of neuromeres recorded in this study, eight (8), was less compared to about ten (10) swellings reported for zebrafish (Kimmel et al. 1995). The lateral wall of diencephalon gave rise to rudiments of eyes, optic primordia, at the early developmental session while at the last part of development, the ventral diencephalon expanded and resulted to primordia of hypothalamus and epiphysis that developed as a *"case"* at midpart of the diencephalic roof; the mid brain primordium then developed to form the dorsal mid brain (optic) tectum and the ventral mid brain tegmentum (Kimmel et al. 1995).

The newly hatched larvae were blind, with sealed mouth therefore needed no exogenous feeding in early few days of life because they were hatched possessing largely filled yolk sac. As organogenesis continued, that is formation of barbells, optic, otic, buccal and olfactory etc. adequate care is imminent towards healthy larvae production. The transition from translucent nature of the larvae to opaque through body pigmentation served as protection. The body pigmentation of melanophore - that is a neural crest-derived cell containing black melanin pigment (Kimmel et al. 1995) firstly started around the eyes and later concentrated heavily on the cranial region before it progressively covered the body. The method of spreading of the pigmentation was the same for *P. maculatus* though it possessed chromatophores body cell pigmentation (Buzollo et al. 2011). Pigmentation has been essential in the taxonomy of species identification (Meijide and Guerrero 2000; Buzollo et al. 2011). The simultaneous development of barbell with its taste bud, removal of epicanthus off the eyes, increase in mouth gaping and gradual depletion of the yolk prepared the larvae for adaptation and active exogenous feeding. Our results indicate that larvae of *H. bidorsalis* are highly photophobic like *S. parahybae* (Caneppele et al. 2009; Honji et al. 2012); and very active at night than day time like their congener, *H. longifilis* (Anselme et al. 2008). These attributes have to be cared for during feeding programmes or nutritional studies for better performance, hence feeding in the dark is recommended. The low/dim light intensity or dark environment reduces stress on the larvae and consequently improves their development (Appelbaum and Mcgeer 1998; Honji et al. 2012). Feeding prior to utilization of barbells to search for food influences high survival and growth performances (Kamler 1992; Haylor and Mollah 1995; Olaniyi and Omitogun 2013). Moreover, as cannibalism has been established to be unavoidable in many fish species, *ad libitum* feeding that is, availability of feed at all times, shorter feeding interval, and low stocking density among others (Howell et al. 1998; Honji et al. 2012) as employed in this study is the best way to greatly reduce it. Cannibalism has been established in 36 out of 410 teleost families and several unreported families (Smith and Reay 1991; Honji et al. 2012). Therefore, the knowledge of feeding practices and behaviour that will meet the nutritional requirement of fish larvae is essential to have successful fish breeding and aquaculture programme.

Furthermore, no fin bifurcation to dorsal and adipose fins, which is the unique identification of *Heterobranchus spp* was observed prior to exogenous feeding of the larvae, but was revealed at the third and half weeks post-hatch with no black spot at the tail end of adipose fin. The absence of black spot at the tail end of *H. bidorsalis* is one of the features that distinguishes the species from its congeners (Reed et al. 1967; Teugels et al. 1990).

## Conclusions

This study for the first time investigated and registered significant morpho-sequential developmental stages in the ontogeny and organogenesis of *H. bidorsalis*. The study pointed out polster and tail bud as morphological markers in the identification of the cranial and caudal parts of the developing embryo respectively. In addition, artificial propagation of the species is the best as this study discovered the unique mode of eclosion that has great effect on water quality. The present knowledge recommends *ad libitum* feeding under low/dim light intensity or dark environment as appropriate for the larvae being photophobic. These important features must be highly cared for in feeding regime to have successful propagation of the species. The data from this study contribute immensely to the understanding of the biology of *H. bidorsalis* species for aquaculture, artificial propagation and also have implications for genetic manipulation, ecological and conservational researches.

## Abbreviations

EVL: Enveloping layer
YSL: Yolk syncytial layer.
